# Graduate Study on the Continent—Germany

**Published:** 1908-05-02

**Authors:** 


### GRADUATE STUDY ON THE CONTINENT.—GERMANY.

### II.—BERLIN AS A CENTRE FOR MEDICAL STUDY.

Of all German university towns Berlin offers perhaps the most facilities for study for the graduate, especially for the "foreign graduate" who comes to Germany in search of "special courses." The population of the city is of the most varied kind, and medically Berlin taps the surrounding parts to a greater extent than is the case in London and Paris. The large metropolitan hospitals are always replete with interest, and the city itself swarms with private clinics and polyclinics which are available for the industrious student. So far as hospitals are concerned, Berlin can now boast of possessing the largest and, in some points, the best-equipped hospital in the world—the gigantic Virchow Infirmary, recently completed—and of a number of interesting and well-filled institutions in various parts. At these charities the opportunities for study are numerous and varied. Any "specialty" can be worked at throughout the year, for there is always material and there is always someone able and willing to demonstrate how such material may be best utilised. As a centre for graduate work Berlin is probably unique, in so far that it offers, in its private polyclinics, opportunities for special work which are unobtainable anywhere else.

The private polyclinic is an essentially German institution, and has reached its apotheosis in Berlin, where every main thoroughfare holds one or more of such institutions. The foundation on which it is built is club practice. The lower classes who can afford to pay a nominal sum belong to clubs which have each their special *kranken kasse*, or sick fund, to which is attached one or more medical officers who are paid so much per head. In its essentials, such club practice is similar to that which obtains in England. It differs, however, in this, that each medical officer attached to such a club treats his patients as out-patients, and has consequently a small visiting list; those who are ill enough to be classed as "bed patients" being promptly sent to an infirmary. The private polyclinics are thus out-patient departments which are open to the profession to attend. Every courtesy is shown to the graduate visitor who drops in to these demonstrations, and in many cases it is possible to obtain an assistantship to the professor in charge of the clinic. The assistant has his hands full; he is clerk, house surgeon, and dresser in one; but his cases are more or less selected, and he is responsible only to his chief. The consideration of the merits of the system must be left for a future article. For the present it is only necessary to draw attention to the existence of these polyclinics and the facilities which they offer.

At all the general hospitals exceptional opportunities for graduate work are offered. Each has one or more special departments, and the officers in charge of these departments are indefatigable in arranging courses, lectures, and demonstrations for students. It must be borne in mind that to a very large extent the aim of all these courses is to give the student the fullest opportunity for practical work. The instruction in medicine and surgery is thoroughly practical. It has been alleged by those who have a superficial acquaintance with German hospital methods that there is very little clinical or bedside teaching. The statement is absolutely devoid of truth. It is true that there is very little of the kind of teaching which one meets with at London hospitals. The demonstrator does not explain to his class the meaning of the normal heart-beat, nor explain that the knee-jerk is not a true reflex, nor draw up a list of the normal blood constituents, nor explain the tests for albumen, indican, or sugar. All this the student is supposed to know. The standard required for a pass in preliminary subjects is higher here than with us, and the student upon entering the wards knows his applied anatomy and physiology fairly well. What he is taught in the wards is purely practical—the diagnosis, prognosis, and treatment of disease, with perhaps a superfluity of pathology. To pass his final examinations he has to depend largely upon himself, for there is less examination teaching than in England. The demonstrations are far less dogmatic and aphorismal than with us, and the student is left very much alone to find out things for himself.

Necessarily preliminary considerations as to cost of study, living expenses, books, etc., will have to be weighed by the graduate who desires to devote a part of his time to study in Berlin. It may at once be stated that although Berlin, in point of expense, compares unfavourably with smaller German university towns, it does so very favourably indeed with other large Continental centres for medical study. The graduate may either live *en pension* or get rooms without board. The former method, which for those in active study is perhaps to be recommended, involves an outlay of from £4 to £8 per mensem. Single bed-sitting-rooms in the neighbourhood of the large clinics may be rented from 30 to 40 marks per mensem, according to situation. Rooms on the first floor or on the ground floor are relatively 10 per cent. dearer than those on the second or third floors. Attendance, boots, lighting, and breakfast are usually included in the monthly price; but washing is, of course, an extra. In any case, the graduate should have a clear and definite arrangement, preferably in writing, as to his liabilities and privileges, otherwise he may find at the end of his term that he has to pay for more extras than he has bargained for. The lodger usually receives two enormous keys, the one of the common house door and the other his special room key, and it is usually advisable to carry a small electric pocket lamp, since the common staircase is generally without a light, and its navigation when returning home late is by no means easy. The attendance is almost without exception good.

If the student wishes to be as independent as possible, it is advisable to hire a room and to arrange for breakfast only with the landlady, all other meals being taken out. Berlin abounds with small restaurants at which fairly satisfactory meals may be obtained at very cheap rates. Such a course is much to be preferred, especially in cases where the student is attached to some institution and has no chance of being absolutely regular in his meal times. The cost of "boarding out" is from £1 15s. to £2 weekly when all meals are taken at a good restaurant *à la carte*, but this expense may be cut down to half when the cheaper restaurants are selected or when an arrangement is made at some particular house.

The expenses of study vary, of course, with the specialty which the student desires. Instruments are, on the whole, cheap; books, on the contrary, are very expensive in comparison with English prices. There are, however, numerous second-hand bookshops, many of them making a speciality in medical books, at which text-books and monographs may be obtained 50 per cent. below cost price, and usually in excellent condition. Then, too, there are various medical reading-rooms which are open to students on payment of a small fee; and lending libraries, similar to the well-known library of Mr. Lewis in Gower Street, which lend out text-books on payment of a small subscription.

The courses are usually given at very low fees; in a list of courses which has just been issued I find that the fees range from 10 to 100 marks, the average working out at approximately 32 marks each. For the fees charged the majority of courses are exceptionally good value. Living expenses may be calculated as being about equal to those in London, while expenses of study are at least 50 per cent. lower. There are several post-graduate associations that cater for the "practising physician," and the newcomer cannot do better than refer to one of them for all particulars. For English-speaking students the excellent Anglo-American Medical Association of Berlin (Office 105 Friedrichstrasse) is a model of its kind.

Among the more important professional societies in Berlin are the following: Berliner Medizinische Gesellschaft, the largest and most important medical society of the city, meets on Wednesday evenings at half-past seven in the Langenbeck Haus, Ziegelstrasse. The Association issues the "Berlin. Medizinisch. Wochenschrift." Membership is open to foreign doctors, if properly proposed and balloted for, subscription 20 marks, no entrance-fee. Professional men temporarily resident in Berlin are granted a guest ticket, which gives the privileges of honorary membership, admission to meetings, use of the fine reading room, etc. No fee. Application for guest ticket to be made in writing to Herr Geheimrat Professor Dr. Ewald, the Honorary Librarian, at his residence, Rauchstrasse 4, Berlin W. In all cases a stamped and addressed envelope should accompany the application. The meetings of the association are exceedingly interesting, and graduates are advised to attend them. Verein für Innere Medizin: meets on Monday evenings at 8 o'clock in Room B. Architektenhaus, Wilhelmstrasse 92, 93. Library open 12-8 at Schöneberger Ufer 11. Berliner Opthalmologische Gesellschaft: meetings Thursday evenings at 8, at the Royal Eye Clinic, Ziegelstrasse 5-9. Laryngological Society, meetings Fridays at 7.30 P.M., Luisenstrasse 13 a. Clinical demonstrations and discussions are given by the staff societies of the various large hospitals. That of the Charité meets irregularly; that of the Friedrichshain Hospital meets at the hospital on Thursdays at 8 P.M. Suburban societies are the Rixdorfer and Charlottenburger Societies, which meet on Mondays and Thursdays respectively. The graduate is advised to apply to Herr Eugen Grosser, Wilhelmstrasse 121 SW. for a copy of the "Berliner Anzeiger (Rothes Blatt)," which is sent regularly and gratis to all medical men, and which gives details of all professional meetings to be held during the ensuing week.

